# Surgical Complications in Conventional and Laparoscopic Cholecystectomy in an Ecuadorian Reference Hospital

**DOI:** 10.7759/cureus.107292

**Published:** 2026-04-18

**Authors:** Carlos Andres Yepez Salgado, Zully Mayra Romero Orellana, Marco Vinicio Moreno Rueda, Telmo Vinicio Sarango Ochoa, Yesenia Liliana Chandi Japon, Dayra Alejandra Imbaquingo Sarmiento

**Affiliations:** 1 Teaching and Research Unit, Hospital Provincial General Docente Riobamba, Riobamba, ECU; 2 Birmingham Clinical Trials Unit, University of Birmingham, Birmingham, GBR; 3 Department of Medical Direction, Hospital Provincial General Docente Riobamba, Riobamba, ECU; 4 Office of the Dean, Universidad Nacional de Chimborazo, Riobamba, ECU; 5 Department of General Surgery, Hospital Provincial General Docente Riobamba, Riobamba, ECU; 6 Department of General Practice, Pungala Health Centre, Riobamba, ECU

**Keywords:** cholecystectomy, cholecystitis acute, gallstones, general surgery, laparoscopic cholecystectomy

## Abstract

Cholecystectomy is a very common surgical intervention that consists of the removal of the gallbladder, especially when complications such as cholecystitis exist. The laparoscopic technique has been associated with greater postsurgical benefits; however, no local research has been conducted comparing these two techniques, considering that in poor-income countries, there are hospitals that do not have the technological and economic resources available to perform laparoscopic surgeries. This project aimed to determine the differences between open versus laparoscopic cholecystectomy, in terms of complications, in patients in the general surgery service from January to June 2024 at the Hospital Provincial General Docente Riobamba, Ecuador. We present an original, observational, cross-sectional, retrospective, quantitative study, with a descriptive and inferential approach. Of a total sample of 107 patients, 69 (64.5%) underwent conventional cholecystectomy and 38 (35.5%) underwent laparoscopic cholecystectomy, of whom the main pathology was cholelithiasis in 100 (93%) cases. A total of 12 (11%) patients presented complications, and the most frequent were biliary leakage and injury to a nearby organ; 26 (24%) had comorbidities, predominantly type 2 diabetes and hypertension. There was insufficient evidence to determine differences between open versus laparoscopic cholecystectomy with the occurrence of surgical complications (p > 0.05). Emergency surgery and the presence of comorbidities conferred a higher probability of surgical complications (OR: 3.53, 95% CI: 1.029 - 12.08, p = 0.049 and OR: 3.75, 95% CI: 1.09 - 12.89, p = 0.028, respectively). It is concluded that there are no differences in complications regarding the type of cholecystectomy (open or laparoscopic), but there were differences in the presence of comorbidities and whether the surgery was an emergency procedure.

## Introduction

Cholecystectomy is a very common surgical intervention that consists of the removal of the gallbladder when it presents lithiasic, infectious, or neoplastic pathologies [1]. More than 50,000 cholecystectomies are performed annually in the United Kingdom, and in the United States, the figure reached approximately 800,000 procedures, at a cost of almost 6,500 million dollars each year [2]. Likewise, in South America, it is reported that annually between 5% and 15% of the inhabitants must be operated on for gallbladder pathologies [3]. In Ecuador, gallbladder diseases are very common morbidity conditions, with cholelithiasis being the pathology that represents the first source of morbidity and the third cause of surgical intervention, with a prevalence of 71% in women and 29% in men [4].

The incidence of cholelithiasis varies depending on age, gender, ethnicity, diet, and even geographical location [5]. It is considered one of the main causes of hospital admissions, associated with a significant health and economic burden [6,7]. Generally, most cases present as asymptomatic, in which cholecystectomy is not indicated; however, prophylactic surgical intervention is usually considered in patients with factors that predict a more serious course in the future [8,9]. Thus, cholecystectomy in biliary lithiasis is used only when complications appear (cholecystitis, pancreatitis, choledocholithiasis, etc.) [10].

The techniques currently available are conventional cholecystectomy (CC) and laparoscopic cholecystectomy (LC) [11]. The CC technique was initially performed through an incision in the right upper quadrant, which was maintained for more than 105 years, but with the passage of time and technological advancements, the technique showed considerable variation and tended to become less invasive [12,13]. This is how, in 1985, laparoscopy emerged in Germany, which spread rapidly to the point of achieving popularity and was the subject of multiple comparative studies [14].

Scientific studies have identified the advantages of the laparoscopic technique, such as reduction in hospital stay, less postoperative pain, better aesthetic results, quick return to work activities, and reduced costs at the country level [15]. However, the use of the open technique is still necessary due to social and economic factors, patient characteristics, and the absence of facilities, instruments, and trained professionals [16].

Additionally, the high incidence of adhesions, difficulty in visualizing structures, or uncontrolled bleeding must be considered [17]. When these circumstances exist, the number of infectious postoperative complications, additional procedures, and readmissions within 30 days increases [18], and this leads to longer postoperative stays and higher morbidity and mortality rates [19,20].

At the Hospital Provincial General Docente Riobamba (HPGDR), only CCs were performed, and as of 2024, the hospital acquired the equipment, materials, and supplies for LCs; therefore, no research has been conducted comparing the impact of these two surgical techniques on patients. Thus, the authors proposed this research aimed to determine the differences between CC and LC in terms of complications, considering an elective or urgent approach, and the clinical and sociodemographic characteristics of patients in the general surgery service from January to June 2024 at the HPGDR.

The specific objectives were to determine the diagnosis requiring cholecystectomies and the percentage of elective and urgent surgeries; to establish the main complications and comorbidities of patients; to determine whether CC differs from LC with respect to the presence of surgical complications; to determine the association between the number of days of hospital stay, amount of trans-operative bleeding, and duration of the operation, with the type of cholecystectomy; and to establish the association between the presence of comorbidities and the type of surgery (elective or urgent), with the appearance of surgical complications.

## Materials and methods

Study design

We present an original, observational, cross-sectional, retrospective, quantitative study, with a descriptive and inferential approach.

Study participants

The total number of patients treated for biliary pathologies in the general surgery service of the HPGDR from January to June 2024 was 175. It was considered to work with the entire population; however, when reviewing the data matrix, a sample of 107 patients were obtained regarding inclusion criteria (patients with complete data of the variables in the matrix, and over 16 years of age) and exclusion criteria (patients with a postoperative diagnosis of open or laparoscopic cholecystectomy who have been operated on in other health institutions). Considering this eventuality and the number of data obtained, the sample calculation was carried out to verify if the sample obtained was sufficient to continue with the analysis. Poisson's approximation reflected that a random sample of 107 individuals is sufficient for the project estimate, with a confidence level of 94%, accuracy of ±6 percentage units, a population percentage that is expected to be around 50%, and estimating a follow-up loss rate of 10%. Therefore, our sample of 107 patients was considered adequate (n = 107). This calculation was performed using the Granmo calculator and can be verified at the following link, which is available free of charge: https://www.datarus.eu/aplicaciones/granmo/. The values ​​should be entered in the "PROPORTIONS, Population Estimation" section.

Ethical considerations

As it is a study that uses secondary sources of anonymized information, and due to its observational methodological nature, it does not represent ethical conflicts because there are no research risks. The hospital does not have an ethics committee for the research, but the formal request was made to the highest authority, and to the Health Care Ethics Committee (CEAS) of the hospital, which granted the respective authorization for the delivery of the information.

The HPGDR trains general practitioners and postgraduates, for which the surgery service for teaching and research purposes implemented a mandatory policy of collecting information from each patient according to the variables described above in the main pathologies, such as appendicitis, biliary pathologies, obstructive abdomen, and fractures, among others. This information is collected by postgraduate doctors and is used for the medical handover, where the case is analyzed with medical specialists (teaching purposes), and the matrices are delivered to the statistics service every month as patients are discharged.

For the collection of information, the patient signs an informed consent form as part of the standard of care, specifying that clinical data will be collected for research and teaching purposes from medical records and surgical protocols, without the inclusion of personal or contact data.

The statistics service collects the information and updates the matrix of hospital discharges daily. The service acts as a safeguard for the information if it could be required for registration and investigative purposes. The information corresponding to the variables was delivered to the principal investigator in an Excel file, completely anonymized, without personal or contact records.

Statistical analysis

The variables studied are as follows: quantitative variables, including age, total bleeding, hospital stay, and duration of the operation; qualitative variables, including sex, elective or urgent surgery, diagnosis, presence of complications, comorbidities, placement of Jackson-Pratt drain, and type of surgery.

To determine the presence of complications, it was considered whether the patient had hemorrhage, defined as any amount of bleeding measured in milliliters that produces hemodynamic instability; bile leaks; infection, either at a surgical or systemic site; or injury to nearby organs [17,18]. This information was recorded in the surgical protocols and in the data matrix provided to the researchers.

The information was filtered according to the inclusion and exclusion criteria, then imported into the STATA 19 statistical package (StataCorp LLC, College Station, TX) for analysis. The statistics used were percentages, measures of central tendency, and dispersion in the univariate analysis. For multivariate analyses, odds ratios (OR), logistic regression, Fisher's exact test, T-test, and chi-squared tests were performed. Each value had 95% confidence interval (95% CI), and statistical significance was determined by a p-value of less than 0.05.

Reporting and detection bias are present due to observational and cross-sectional design; however, it is significantly reduced because the information is collected by medical professionals and statistical technicians, not by researchers. Selection bias is minimal since the calculated sample size was met.

The results were presented in accordance with the Strengthening the Reporting of Observational Studies in Epidemiology (STROBE) guidelines for cross-sectional studies.

## Results

Of the total data analyzed (n = 107), 84 were women (78.5%, 95% CI: 71% - 86%), and 23 were men (21.5%, 95% CI: 14% - 29%). The mean age, regardless of sex, was 41 years (95% CI: 38 - 44), with a minimum of 19 years and a maximum of 83 years. Regarding the type of surgery, 69 (64.5%, 95% CI: 55% - 74%) underwent open CC and 38 (35.5%, 95% CI: 26% - 45%) underwent LC.

Table 1 presents the descriptive results of each additional variable studied in all cholecystectomized patients (n = 107), according to sex distribution.

**Table 1:** Descriptive analysis of patients undergoing gallbladder surgery by sex. * P-value determined by T-test.

Variable	Men	Women	p-value
				n	23 (21.5%)	84 (78.5%)	-
Surgical approach			
Laparoscopic cholecystectomy	5 (21.7%)	33 (39.3%)	0.119
Conventional cholecystectomy	18 (78.3%)	51 (60.7%)	
Type of surgery			
Emergency	8 (34.8%)	26 (31.0%)	0.727
Elective	15 (65.2%)	58 (69.0%)	
Complications			
No	20 (87.0%)	75 (89.3%)	0.754
Yes	3 (13.0%)	9 (10.7%)	
Comorbidities			
No	17 (73.9%)	64 (76.2%)	0.821
Yes	6 (26.1%)	20 (23.8%)	
Required Jackson-Pratt drain			
No	16 (69.6%)	75 (89.3%)	0.019
Yes	7 (30.4%)	9 (10.7%)	
Age	44 (15.447)	40 (16.478)	0.228*
Surgical indication			
Cholecystitis	0 (0.0%)	7 (8.3%)	0.152
Cholelithiasis	23 (100.0%)	77 (91.7%)	
Transoperative bleeding (mL)	60 (59.2)	34.1 (60.1)	0.069*
Hospital stay (days)	4.1 (2.1)	4.2 (2.7)	0.963*
Duration of surgery (minutes)	94.4 (40.6)	89 (40.7)	0.571*

Table 1 reported that elective surgery exceeds emergency surgery by 39 (36%) patients; however, in general, 12 (11%) presented complications, 16 (15%) required placement of a drainage device, and an average of less than one week of hospitalization. Additionally, there were differences (p = 0.019) regarding the placement of the drain when comparing sex, regardless of the type of surgery.

Table 2 presents the baseline table for each variable compared to CC and LC.

**Table 2:** Baseline table for conventional cholecystectomy (CC) and laparoscopic cholecystectomy (LC). * P-value determined by T-test.

	LC, n (%/SD)	CC, n (%/SD)	p-value
N	38 (35.5%)	69 (64.5%)	
Sex			
Men	5 (13.2%)	18 (26.1%)	0.119
Women	33 (86.8%)	51 (73.9%)	
Age (years)	44.1 (16.8)	39.3 (15.9)	0.136
Surgery			
Emergency	13 (34.2%)	21 (30.4%)	0.688
Elective	25 (65.8%)	48 (69.6%)	
Complications			
No	33 (86.8%)	62 (89.9%)	0.636
Yes	5 (13.2%)	7 (10.1%)	
Comorbidities			
No	26 (68.4%)	55 (79.7%)	0.193
Yes	12 (31.6%)	14 (20.3%)	
Jackson-Pratt drain			
No	33 (86.8%)	58 (84.1%)	0.699
Yes	5 (13.2%)	11 (15.9%)	
Surgical indication			
Cholecystitis	7 (18.4%)	0 (0.0%)	<0.001
Cholelithiasis	31 (81.6%)	69 (100.0%)	
Hospital stays (days)	3.7 (2.1)	4.5 (2.8)	0.178*
Transoperative bleeding (mL)	32.4 (32.9)	43.7 (71.4)	0.357*
Duration of surgery (minutes)	92.2 (44.0)	89.1 (38.8)	0.713*

Table 3 reports a strong lack of evidence (p > 0.05) to determine differences between sex, type of surgery, complications, comorbidities, placement of drainage, hospital stay, bleeding, although on average, CCs had a quantity greater (10 mL) than LC, and the duration of the surgery, which in turn does not imply a clinically important difference. The surgical indication variable did show strong evidence of association (p < 0.001), highlighting that no patient with cholecystitis underwent CC.

**Table 3:** Odds ratio of complications in open and laparoscopic surgeries. Number of valid cases: 107.

OR of complications versus type of surgery (CC/LC)				
Complications	OR	95% CI		p-value
		Lower	Upper	
Conventional surgery	0.75	0.219	2.531	0.636
OR by logistic regression of complications versus type of surgery, adjusted for surgical indication				
					Complications	OR	95% CI		p-value
Lower	Upper								
Conventional surgery	1.05	0.25	4.37	0.943
Cholelithiasis	0.26	0.035	2.03	0.203

Complications are distributed according to the following number of cases: two (2%) infections, two (2%) injuries to nearby organs, seven (6%) bile leaks, and one (1%) hemorrhage.

Regarding comorbidities, six (6%) had type 2 diabetes mellitus, six (6%) had high blood pressure, one (1%) had hypothyroidism, one (1%) had obesity, and 12 (11%) corresponded to the sum of other pathologies.

Multivariate analyses

Considering the reported complication rate of 12 (11%) patients, and despite the lack of evidence to determine differences between CC and LC, it was decided to calculate the OR and adjusted OR for the surgical indication variable, which was the only one that showed evidence of association. These results are presented in Table 3.

Undergoing CC determines a 25% lower chance of having complications; however, there is a wide range of uncertainty given by the 95% CI, which is corroborated by the p-value, which reports the lack of evidence to accept the percentage obtained (p > 0.05). Additionally, the OR value, when adjusted for the surgical indication, changed, indicating a 5% higher probability of complications. This ambiguity in the results is reinforced by a p-value that demonstrates strong evidence (p = 0.943) of no difference between CC and LC in terms of surgical complications.

Table 4 is presented to establish the association between the presence of comorbidities and complications.

**Table 4:** Odds ratio (OR) of presence of complications in patients with comorbidities. Number of valid cases: 107.

Complications	OR	95% CI		p-value
		Lower	Upper	
Presence of comorbidities	3.75	1.091	12.886	0.036

Table 5 shows the association between the type of surgery (elective or emergency) and the presence of complications.

**Table 5:** Association between the type of surgery and the presence of complications. Number of valid cases: 107.

Complications	OR	95% CI		p-value (Fisher)	
		Lower	Upper		
Emergency surgery	3.53	1.029	12.077	0.045	

This result shows that emergency surgeries confer a 3.53 times greater probability of having surgical complications compared to patients undergoing elective surgery. Although there is evidence of this association according to the p-value obtained, this evidence is not strong, and it relates to the confidence interval, which is very wide and close to unity.

## Discussion

The results showed that there is a higher percentage of women who undergo cholecystectomy compared to men. These data are supported by scientific evidence, since worldwide, the prevalence of cholelithiasis is around 6.1%, and approximately 5.4% are men and 7.6% are women [21,22]. It is important to highlight that this pathology occurs more frequently in adulthood, and its prevalence is high even in people over 65 years of age [21]. These data are corroborated by our research, since there was an average age of 41 years, but the range of occurrence of this pathology with surgical need ranges from 19 to 83 years.

For the analyses, it was not possible to have the same proportion of patients undergoing conventional and laparoscopic surgery, i.e., 69 (64.5%) versus 38 (35.5%), respectively; however, robust statistical methods were used to mitigate detection bias.

Based on the univariate analysis, most patients who underwent cholecystectomy between January and June 2024 were elective cases (73, 68%) compared to emergency cases (34, 32%), with cholelithiasis being the main cause of the intervention with 100 (93%) patients, but just 12 (11%) had complications, and 26 (24%) had comorbidities. The main cause of cholecystitis and therefore cholecystectomy is cholelithiasis; in this type of pathology, where cholecystitis is already present, it is necessary to intervene surgically urgently; however, when it comes to cholelithiasis without cholecystitis, treatments are usually expectant with clinical management unless the symptoms worsen [23].

Although most people did not have comorbidities, in our study, the ones that did occur most frequently were diabetes and hypertension, which added up to 12 (12%) cases, followed by obesity and hypothyroidism, with a total of two (2%) people. These results are to be expected since they are the most frequent chronic pathologies worldwide, with diabetes being a disease that confers an individual risk for the appearance of biliary pathologies [10], and both hypertension and hypothyroidism have a combined presentation and association along with cholelithiasis and cholecystitis [21,24,25].

With an average number of four days of hospitalization, an average surgery duration of 60 minutes, and 38 ml of bleeding, the most frequent complication was bile leakage in both types of surgery, with seven (6%) presentations. Controlling bleeding is essential in surgical procedures; however, transoperative bleeding is expected as long as hemodynamics are not affected, considering that additional complications such as bile leaks are frequent [26,27].

The importance of this study, which addresses the main objective, lies in Table 2, which shows strong evidence of no differences between the variables studied regarding the type of surgery (CC or LC), especially when evaluating whether greater complications depend on the surgical approach used. This contradicts the scientific literature that supports LC as the preferred approach due to its lower complication rate [12]. However, in the context of this study, where the availability of laparoscopic towers and supplies is limited due to scarce economic resources nationwide, these results support the continued use of open surgery procedures, which have a similar presentation of complications, bleeding, hospital stay, etc.

Although this study reported that there was a lack of evidence to consider that laparoscopic surgery is superior to conventional surgery with respect to the presence of complications (OR: 0.75, 95% CI: 0.22 - 2.53; p = 0.636), research reports that the overall prevalence of bile duct injury was 0.87% in patients undergoing laparoscopic cholecystectomy and 0.58% in those undergoing open cholecystectomy [28]. In contrast, the evidence reports LC as the gold standard for its lower incidence of complications, but it should be noted that there is a conversion rate from LC to open surgery in cases of surgeon inexperience, obesity, thickening of the gallbladder wall, and higher Parkland classification scores [29]. This result may be due to the inequality in the proportion of patients undergoing conventional and laparoscopic surgery in our sample, the absence of patients with cholecystitis undergoing CC, or to hospital factors such as the expertise and experience of each general surgeon. It is advisable to reinforce this research with longitudinal studies that allow controlling confounding factors and working with the entire population in a follow-up time of at least one year, and to contrast not only the presence of complications, but also the costs generated in open versus laparoscopic surgeries.

Similarly, there was insufficient evidence to determine that conventional versus laparoscopic surgery differed in the days of hospitalization, trans-surgical bleeding, and duration of the operation (p > 0.05). These results differ from recent research showing that LC is the technique of choice, even more so if it is an early LC after percutaneous transhepatic vesicular drainage in acute cholecystitis, which leads to shorter hospital stays and complications [30].

The important results to highlight are that the presence of comorbidities, regardless of disease type, increases the chance of complications in both surgical types (OR: 3.75, 95% CI: 1.091 - 12.89; p = 0.028). Although the CI obtained should be considered to be very broad, the p-value reinforces the result, and the evidence supports this finding due to the high prevalence of chronic diseases involved in the pathophysiology of biliary diseases [25,27]. In addition, emergency surgeries are associated with a higher probability of complications (OR: 3.53, 95% CI: 1.029 - 12.01; p = 0.049), regardless of the CI and the p-value, which are statistically significant, but are very close to the unit. These are expected results because an urgent surgery confers greater complications since there is no possibility of performing pre-surgical check-ups beforehand, in addition to the complications of the disease that are already causing hemodynamic instability, which is why an emergency operation is decided.

According to the study design, it should be clarified that there are biases that must be considered to make conclusions about the differences between CC and LC. One of the main weaknesses is not having a follow-up of each patient that assesses the presence of immediate complications during hospitalization and at discharge. In the same way, the heterogeneity of the participants includes important confounding factors that could be controlled in prospective cohort studies.

Despite the limitations, our study has important strengths. The main strength lies in the fact that it is the first study carried out in the HPGDR that compares the two surgeries, and important epidemiological data are presented that mark the starting point to continue with the realization of future and more solid research. It is important to mention that the appropriate statistical analyses were used depending on the type of variables, the required sample was met, and there were no ethical conflicts or risks for the patients.

The main recommendation is to carry out studies with follow-up, minimizing selection and detection bias, confounding factors, and reverse causality.

## Conclusions

It is concluded that the main diagnostic indication for open or laparoscopic cholecystectomies was cholelithiasis with 100 (93%) cases, versus seven (7%) patients with cholecystitis, of which 73 (68%) surgeries were elective and 36 (32%) were emergency. Twelve patients (11%) presented complications, of which the most frequent was biliary leakage, followed by nearby organ injury and infection. A total of 26 (24%) patients had comorbidities, with type 2 diabetes mellitus and arterial hypertension being the most frequent. There was a lack of evidence to determine differences in the presence of complications between conventional and laparoscopic cholecystectomies, hospital stay, amount of transoperative bleeding, and duration of the operation. However, having comorbidities and undergoing emergency gallbladder surgery confer a higher probability of having surgical complications.
